# Production and characterization of coconut shell charcoal-based bio-briquettes as an alternative energy source for rural communities

**DOI:** 10.1016/j.heliyon.2024.e35717

**Published:** 2024-08-09

**Authors:** John Yirijor, Alice Abigail Tatenda Bere

**Affiliations:** a Department of Materials Science and Engineering, University of Ghana, Legon, Ghana; b Department of Mechanical Engineering, Academic City University College, Haatso, Accra, Ghana

**Keywords:** Fixed carbon, Ash content, High heating value, Moisture content, Ignition time

## Abstract

The increasing demand for sustainable energy solutions has driven interest in the utilization of agricultural residues, such as coconut shells, for bio-briquette production. This study investigates the impact of binder types (cassava and corn) and concentrations (5 wt%, 10 wt%, 15 wt%) on the properties of bio-briquettes made from dried coconut shells with two particle sizes (40 mesh and 60 mesh). The experimental evaluation focuses on several performance indicators, including density, shatter index, percentage moisture content (PMC), percentage volatile matter (PVM), percentage ash content (PAC), percentage fixed carbon (PFC), higher heating value (HHV), ignition time, burning time, and boiling time. The results indicate that briquettes with 10 % fine charcoal cassava binder achieved the highest density of 0.764 g/cm³ due to improved compaction. Briquettes with 15 % coarse charcoal corn binder demonstrated the highest shatter resistance at 96.99 %, reflecting their superior structural integrity. The highest PMC and PVM values were observed in briquettes with 15 % coarse charcoal cassava binder, at 8.13 % and 31.25 %, respectively. Conversely, the highest PAC was 16.34 % for 5 % coarse charcoal cassava binder. Briquettes with 10 % fine charcoal corn binder exhibited the highest PFC of 70.79 % and HHV of 31.51 MJ/kg. Boiling times ranged from 15 min 53 s to 36 min 35 s, with the shortest boiling time for briquettes with 10 % fine charcoal corn binder. The findings highlight the superior mechanical properties and energy performance of bio-briquettes with specific binder concentrations and particle sizes. This study demonstrates the potential of coconut shell bio-briquettes as a viable and sustainable energy source, offering economic and environmental benefits through the effective utilization of agricultural waste and reduction of carbon emissions.

## 1 Introduction

Access to reliable and sustainable energy sources is crucial for societal development, yet remains a significant challenge in many rural areas, including Ghana. Recent reports indicate that the electricity supply in Ghana falls short of the targeted demand, particularly affecting rural communities [1]. This energy gap compels approximately 73 % of Ghanaians to rely on traditional energy sources such as firewood and charcoal for cooking and heating [29], contributing to environmental degradation, deforestation, and greenhouse gas emissions [2]. Globally, about 41 % of households, equating to more than 2.8 billion individuals, depend on solid fuels like coal and biomass for their energy needs [5,30].

Agricultural residues, such as coconut husks, rice straw [3], sugarcane leaves [4], palm kernel shells [5], and peanut shells [31], present promising alternatives as biomass feedstocks for bio-briquettes. These materials offer affordable and sustainable energy solutions [[8], [9]], but their direct use is often impeded by challenges such as low density and high moisture content [6,7]. Cassava, with its adhesive properties and widespread availability, has emerged as an effective binder in bio-briquette production [32]. Cassava-based binders enhance the mechanical strength and combustion characteristics of briquettes, supporting their potential as a viable energy source for rural communities [33].

Coconut shells, abundant in Ghana's coastal regions [10], are currently underutilized despite their significant potential as an energy source [11]. Improper disposal of coconut waste, which can amount to up to 18 tonnes daily in areas like the Madina Municipality, exacerbates environmental issues such as flooding and occasional fires [34]. Utilizing coconut shell charcoal-based bio-briquettes offers a promising solution to these challenges by providing cleaner energy with reduced emissions and a smaller carbon footprint. Previous research has demonstrated the energy potential of coconut shells [35] and highlighted the need for their effective utilization in sustainable energy solutions.

Despite progress in bio-briquette technology, gaps remain in optimizing production methods tailored to local conditions in Ghana. This study aims to address these gaps by investigating the mechanical and combustion properties of bio-briquettes derived from coconut shells. Specifically, the research will focus on optimizing binder compositions and particle sizes to enhance performance and commercial viability in rural Ghanaian contexts. The objectives of this study are to: (1) evaluate the impact of different binder types and concentrations on the properties of coconut shell bio-briquettes; (2) assess the effect of particle size on briquette performance; and (3) provide insights into the practical applications of these briquettes as a sustainable energy source.

The structure of this paper is as follows: Section 2 details the experimental procedures used for briquette preparation and testing. Section 3 presents the results and discusses the findings in relation to the impact of binder types, concentrations, and particle sizes. Section 4 c oncludes with an analysis of the study's implications and potential applications for improving energy sustainability in rural Ghana.

### 1.1 Experimental procedure

**Fig. 1:**
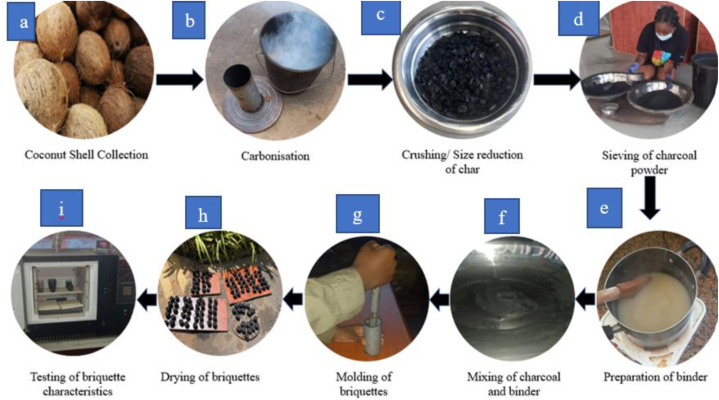
Step-by-step process of briquette production.

The production of coconut shell bio. Briquettes involves several steps, as illustrated in Fig. 1.

#### 1.1.1 Coconut shell collection

Fresh coconut shells were gathered from coconut vendors in the Madina community and air-dried at a temperature of 31 °C for two weeks to lower their moisture content prior to the experiments (Fig. 1a).

#### 1.1.2 Carbonization process

Charring experiments were carried out using a procedure similar to that of Bonsu et al. [5] at the Academic City University College engineering workshop. A makeshift furnace, constructed from a metal bucket, was employed for the carbonization process (Fig. 1b). The metal bucket, with a width of 20 cm at both the topmost and bottommost and a depth of 30 cm, was used to facilitate controlled burning. To ensure a slow and uniform burn, the bottom of the bucket was perforated with a screw (2.11 mm in diameter, 31.25 mm in length). A 15 cm diameter hole was cut into the top of the bucket's lid using a knife. A cylindrical pipe, measuring 29 cm in height and 14.9 cm in diameter, was placed into this hole to serve as a chimney (Fig. 1b). A small amount of dried leaves was utilized to ignite the coconut shells inside the bucket. Once the initial smoke emerged (Fig. 1b), sand was used to cover the sides of the bucket to ensure it remained enclosed. Once the coconut shells were packed into the container, the lid and chimney were secured. The coconut shells underwent controlled combustion, initially producing creamy brown smoke (Fig. 1b). After burning for an hour, the coconut shells were converted into biochar, with the final stage indicated by blue smoke (Fig. 1b), signifying the completion of the carbonization process.

#### 1.1.3 Crushing/size reduction of char

The carbonized coconut shells were crushed into smaller pieces to reduce their size. This step ensures uniformity in the particle size of the char, which is crucial for the subsequent briquetting process (Fig. 1c).

#### 1.1.4 Sieving of charcoal powder

The pervurized char was sieved to obtain fine charcoal powder, with with coarse (40 mesh) and fine (60 mesh) particles (Fig. 1d). Sieving helps in removing larger particles and impurities, resulting in a homogeneous powder suitable for briquette production.

#### 1.1.5 Preparation of binder

A binder was prepared using cassava and corn in varying concentration 5 wt%, 10 wt%, 15 wt%) (Fig. 1e). The binder enhances the cohesiveness of the charcoal powder, aiding in the formation of solid briquettes.

#### 1.1.6 Mixing of charcoal and binder

The experiment involved creating 12 distinct mixtures of charcoal and binders, categorized by the type of charcoal (60 mesh or 40 mesh) and the binder type (cassava or corn) used. Each type of charcoal was mixed with varying concentrations of binders (5 wt%, 10 wt%, and 15 wt%), resulting in a total of 12 unique combinations (as shown in Fig. 1f). This approach was designed to comprehensively assess how different binder concentrations impact the quality and performance of the briquettes.

#### 1.1.7 Molding of briquettes

The mixture of charcoal powder and binder was molded into briquettes using a manual press. The sample –binder mixtures were poured into a hand mold, and a hammer was used to manually compress the mixture, creating more compact briquettes with heights 4.66 and 4.5 mm Fig. 1g). for each concentration, 10 briquettes were produced, giving a total of 120 briquettes.

#### 1.1.8 Drying of the briquettes

The molded briquettes were sun –dried for seven days to reduce their moisture content (Fig. 1h). proper drying is essential to improve the mechanical strength and combustion properties of the briquettes.

#### 1.1.9 Testing of Briquette characteristics

The dried briquettes were tested for various properties, including density, shatter index, percentage moisture content (PMC), percentage volatile matter (PVM), percentage ash content (PAC), percentage fixed carbon (PFC), higher heating value (HHV), ignition time, and burning time, following ASTM standards for specific analyses (e.g., ASTM E711-17 for HHV determination, ASTM D3172-13 for proximate analysis) (Fig. 1i). Testing helps in evaluating the performance and quality of the briquettes, ensuring they meet the desired standards for use as an alternative energy source.

### 1.2 Determination of physical properties

#### 1.2.1 Density

The mass, height(h), and diameter (d), of 5 of each briquette sample were measured using an electronic balance and Vernier callipers. The volume, V of the briquettes was calculated using Equation (1) for the volume of a cylinder.

(1) $$V=\pi \frac{d^{2}}{4}\times h$$

The density, ρ, of the briquettes was calculated using Equation (2) and the results are shown in Fig. 5.

(2) $$\rho =\frac{mass}{volume}$$

#### 1.2.2 Shatter index

The briquettes' resilience or shatter index was determined by dropping the briquette from a height of 1.5 m onto a flat steel plate, which was done four times. The percentage weight loss and shatter resistance were calculated from Equations (3), (4)) [12]. The results are shown in Fig. 6.

(3) $$\%\;Weight\;loss=\frac{initial\;weight\;before\;shattering\;-weight\;after\;shattering}{initial\;weight\;before\;shattering}\times 100$$

(4) Shatterresistance=100−%Weightloss

#### 1.2.3 Percentage moisture content

To calculate the percentage moisture content (PMC), 2 g of the briquette sample was weighed in a crucible of known mass and placed in an oven set at 105 °C ± 5 °C for 1 h allowed us. After the crucible and its contents had cooled, they were taken out of the oven and reweighed. Once the weight after cooling became consistent, the procedure was repeated with the crucible in the oven, and the result was recorded as the final weight, W2. Fig. 4 displays the results of the PMC calculation using the sample's initial weight, W1, as derived from Equation (5) [13].

(5) $$PMC=\frac{W1-W2}{W1}\times 100$$

#### 1.2.4 Percentage of volatile matter

The percentage volatile matter (PVM) was calculated by placing 2 g of broken briquettes in an oven set to a temperature of 550 °C ± 5 °C for 10 min. Three more times for each sample were then completed this method. The PVM was then calculated using Equation. (6) [13].

(6) $$PVM=\frac{M2-M3}{M2-M1}$$

where: M1 = weight of the empty crucible, M2 = weight of the crucible with the sample before heating and M3 = weight of the crucible with the sample after 10 min in the oven at 550 °C.

#### 1.2.5 Percentage ash content

The empty crucible was weighed and designated M1. An amount of 2 g of the briquette samples was placed in a furnace to burn completely at 820 °C for 2 h. The crucible was then weighed with the sample and marked M2 before being placed in the furnace. The crucibles were then placed in the furnace until the samples were reduced to ash. The residue was weighed on an electronic balance and its combined weight with the crucible was designated M3. The percentage ash content (PAC) was then calculated from Equation (7) [12].

(7) $$PAC=\frac{M3-M1}{M2-M1}\times 100$$

#### 1.2.6 Percentage fixed carbon

The percentage fixed carbon (PFC) was calculated using Equation (8) [13].

(8) PFC=100%−(PMC+PVM+PAC)

where: PMC = Percentage moisture content, PVM = Percentage volatile matter, PAC = Percentage ash content.

#### 1.2.7 Higher heating value

The Higher Heating Value (HHV) was calculated using Equation (9) [14].

(9) HHV=2.326(147PFC+144PVM)

### 1.3 Determination of combustion properties

#### 1.3.1 Ignition time

A Bunsen burner was used to ignite each sample at the edge of its base. The ignition time was determined by using a timer to time how long it took for each briquette to ignite. The stopwatch was started the moment the briquette contacted the burner's flame and stopped the moment the base of the briquette was fully ignited. This process was done three times and the average taken.

#### 1.3.2 Water boiling test

This experiment compared the cooking performance of several briquettes and derived how long each briquette took to boil an identical amount of water under identical circumstances. Thus, 200 ml of water was boiled with 125 g of each briquette sample using a small saucepan and a coal pot. The stopwatch was started the moment the saucepan was placed on the coal pot and stopped the moment the water started to boil.

#### 1.3.3 Burning time

To obtain the burning time, the stopwatch was started the moment the briquette was ignited and stopped when the briquette was turned completely into ash.

## 2 Results and discussion

### 2.1 Physical and combustion characteristics of coconut shell briquettes

The physical and combustion characteristics of the coconut shell briquettes were recorded in the tables below. The sample codes 40 mesh charcoal particles with maize/corn binder (CM), 40 mesh charcoal particles with cassava binder (CC), 60 mesh charcoal particles with maize/corn binder (FM), and 60 mesh charcoal particles with cassava binder (FC), respectively. The characterization of the briquettes was done based on ASTM standards.

### 2.2 Analysis of results

#### 2.2.1 Effect of particle size, binder concentration and type of binder on density of briquettes

**Fig. 2:**
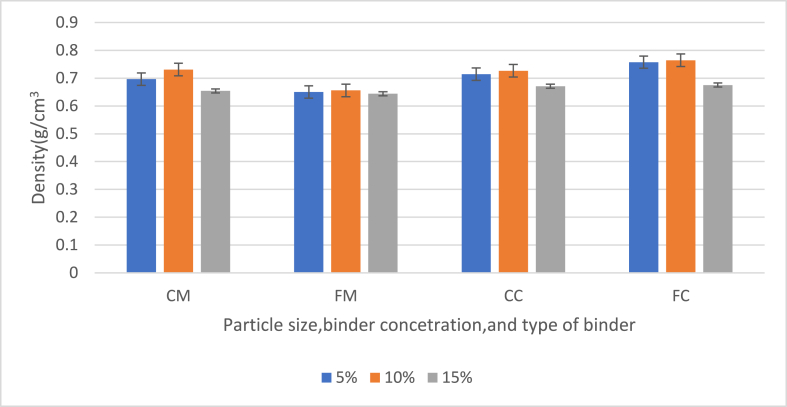
Effect of particle size, binder concentration and type of binder on the density of briquettes at different binder concentrations (CM = 40 mesh charcoal particles with corn binder, FM = 60 mesh charcoal particles with corn binder (FM), CC = 40 mesh charcoal particles with cassava binder, FC = 60 mesh charcoal particles with cassava binder).

Fig. 2 shows the effect of particle size, binder concentration and type of binder on of the density of briquettes with 5 %, 10 % and 15 % binder contents.

The highest density was for 10 % fine particles with cassava binder at 0.764 g/cm^3^ and the lowest was for 15 % fine particle with maize binder with 0.644 g/cm^3^. Generally, the FM samples had low densities, whereas FC samples had higher densities. The results indicate that binder ratios of 10 % produced the highest densities. The briquettes produced in this study had higher densities than wood charcoal, implying that denser briquettes will burn more quickly.

#### 2.2.2 Effect of particle size, binder concentration and type of binder on shatter resistance of briquettes

**Fig. 3:**
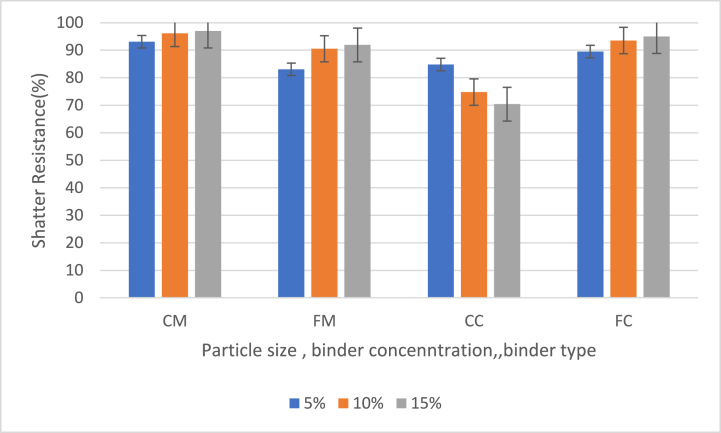
Effect of particle size, binder concentration and type of binder on shatter resistance of briquettes at 5 %, 10 % and 15 % binder contents (CM = 40 mesh charcoal particles with corn binder, FM = 60 mesh charcoal particles with corn binder (FM), CC = 40 mesh charcoal particles with cassava binder, FC = 60 mesh charcoal particles with cassava binder).

Fig. 3 shows the effect of particle size, binder concentration and type of binder on shatter resistance of briquettes on the average shatter resistance of briquettes at 5 %, 10 % and 15 % binder contents.

**Fig. 4:**
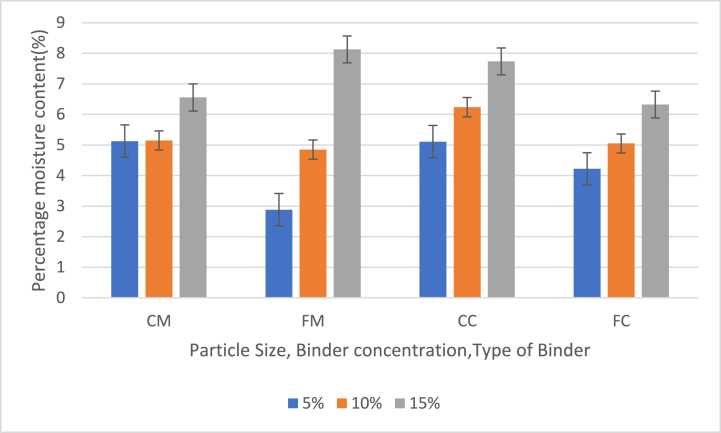
Effect of particle size, binder concentration and type of binder on Percentage Moisture Content (PMC) of briquettes at 5 %, 10 % and 15 % binder contents (CM = 40 mesh charcoal particles with corn binder, FM = 60 mesh charcoal particles with corn binder (FM), CC = 40 mesh charcoal particles with cassava binder, FC = 60 mesh charcoal particles with cassava binder).

**Fig. 5:**
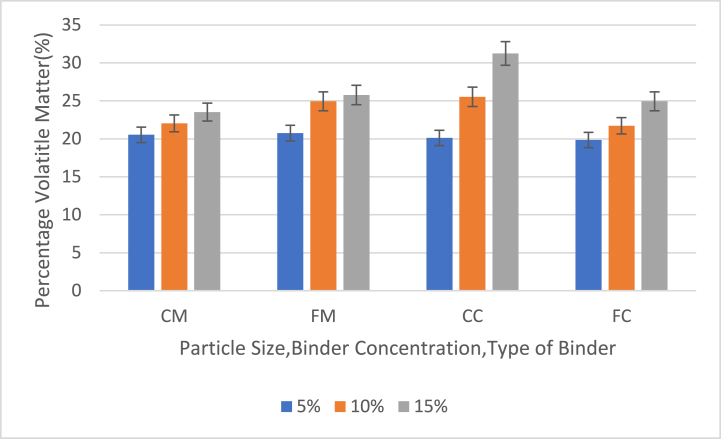
Effect of particle size, binder concentration and type of binder on percentage volatile matter (PVM) values and graph of the PVM of briquettes with 5 %, 10 % and 15 % binders (CM = 40 mesh charcoal particles with corn binder, FM = 60 mesh charcoal particles with corn binder (FM), CC = 40 mesh charcoal particles with cassava binder, FC = 60 mesh charcoal particles with cassava binder).

**Fig. 6:**
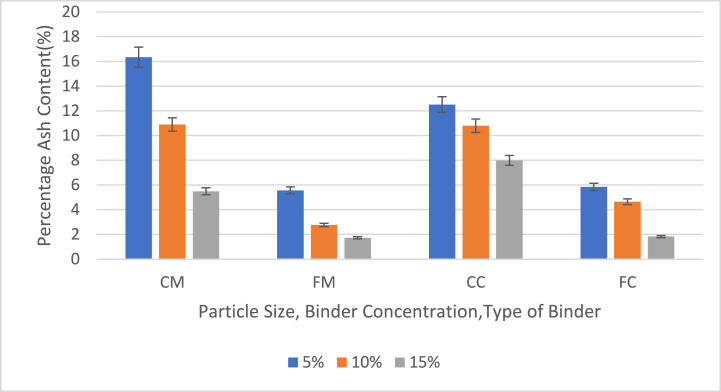
Effect of particle size, binder concentration and type of binder on percentage ash content (PAC) of briquettes briquettes with 5 %, 10 % and 15 % binders (CM = 40 mesh charcoal particles with corn binder, FM = 60 mesh charcoal particles with corn binder (FM). CC = 40 mesh charcoal particles with cassava binder, FC = 60 mesh charcoal particles with cassava binder).

The shatter resistance was generally high for all the briquette samples, which shows that the briquettes should be able to withstand handling and transportation without breaking apart. The average shatter resistance recorded for the samples was 95.39 % for CM samples, 88.50 % for FM samples, 76.68 % for CC samples and 92.66 % for FC samples.

The CM samples had generally higher shatter resistance with an average of 95.39 % and CC samples had generally lower shatter resistance with an average of 76.68 %. The highest recorded shatter resistance was from the CM sample with 15 % binder and a shatter resistance of 96.99 % whilst the lowest recorded shatter resistance was from the CC sample with 15 % binder and a shatter resistance of 70.4 %.CM and FC samples had higher shatter resistances of 95.39 % and 92.66 %

Yirijor et al. [15] recorded mean durability indices ranging from 98.20 %–98.58 % for coconut husk and corncob briquettes mixed at ratios of 80:20, 60:40, 40:60 and 20:80.

#### 2.2.3 Effect of particle size, binder concentration and type of binder on percentage moisture content (PMC) of briquettes

Fig. 4 shows the effect of particle size, binder concentration and type of binder on percentage moisture Content (PMC) of briquettes at 5 %, 10 % and 15 % binder contents.

The briquette samples produced average moisture content with the lowest of 5.20 % from FC and the highest value 6.36 % from CC samples. The fine particle with maize binder samples had the highest average percentage moisture content. The moisture content was generally low for briquettes with the highest value being 8.13 % from FM samples with 15 % binder and the lowest being 2.88 % also from FM samples with 5 %. Binder contents of 15 % had higher MC and binder contents of 5 % had lower MC across all samples, indicating that there was increased moisture content with increased binder contents which agreed with Aransiola et al. [16]. The MC values were within the range of values obtained by Yuliah et al. [17]. The findings support the recommendation Pallavi et al. [18] that good quality briquettes should have a moisture content in the range of 5%–10 %. The results also agreed with Ajimotokan et al. [19] and Akowuah et al. [13]. Fig. 4 shows that fine particle briquettes with a lower maize binder concentration had low MC, and fine particles with a high Maize binder concentration had high MC. Briquettes with low MC were able to ignite during burning and are expected to have HHV. The higher MC would cause the briquettes to produce smoke with a low burning rate.

#### 2.2.4 Effect of particle size, binder concentration and type of binder on percentage volatile matter (PVM) of briquettes

Fig. 5 shows the effect of particle size, binder concentration and type of binder on percentage volatile matter (PVM) of briquettes with 5 %, 10 % and 15 % binder contents.

The volatile matter of all briquette samples was below 35 % with the highest volatile matter being 31.25 % from CC samples with 15 % binder. The lowest volatile matter was 19.85 % from FC samples with 5 % binder contents. The average volatile matter for the samples across all binder contents was 22.032 % for CM samples, 23.82 % for FM samples, 25.63 % for CC samples and 22.17 % for FC samples. Most of the briquette samples fell within the acceptable range for PVM of 10% - 25% for quality briquettes. This indicates that the briquettes produced can easily ignite, burn rapidly, and have a proportionate increase in flame length [28]. This indicates that CC samples had a higher percentage of volatile matter than the other samples whereas CM samples had a lower PVM than the other samples. The data also indicates that briquettes made with corn binder had lower PVM compared to briquettes made with cassava binder. Binders of 15 % produced higher PVM and binders of 5 % produced lower PVM, indicating that PVM increases as the binder was increased. The PVM obtained was within the range of 25 %–35 % obtained by Mfomo et al. [20] indicating that the briquettes will not produce smoky frames during burning. The values obtained from each sample were far below the value obtained by Epesse Misse et al. [21] and Akowuah at el [13]. The accepted value of PVM for agro wastes should be less than 40 % to enhance the briquette's characteristics [22,23].

#### 2.2.5 Effect of particle size, binder concentration and type of binder on percentage ash content (PAC) of briquettes

Fig. 6 shows the effect of particle size, binder concentration and type of binder on percentage ash content (pac) of briquettes with 5 %, 10 % and 15 % binder contents.

The ash content of the briquette samples varied from 16.34 % to 1.72 %. CM briquettes exhibited the highest PAC at 5 % binder, while FM samples had the lowest PAC at 15 % binder. The average PAC values were 10.91 % for CM, 3.35 % for FM, 10.43 % for CC, and 4.11 % for FC samples. Briquettes made from fine charcoal particles (FM, FC) generally had lower ash content compared to those made from coarse particles (CM, CC). Additionally, increasing the binder content resulted in a reduction in ash content. The ash content of the coconut shell charcoal-based briquettes fell within the acceptable range for solid biomass briquettes (5–40 wt%) [40]. High ash content typically leads to more combustion remnants and a lower heating value, which affects heat transfer and oxygen diffusion during combustion [41]. Excessive ash content can hinder effective combustion. In contrast, Yirijor et al. [15] reported a mean ash content of 3.17 %–5.60 % for briquettes made from coconut husk and corn cob. The lower PAC observed in fine particle briquettes suggests that fewer impurities are present, allowing a greater proportion of the mass to contribute to energy release during combustion, thereby enhancing the heating value.

#### 2.2.6 Effect of particle size, binder concentration and type of binder on percentage fixed carbon (PFC) of briquettes

**Fig. 7:**
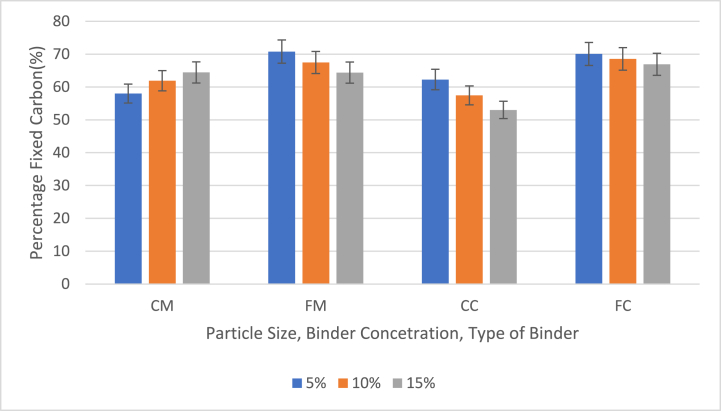
Effect of particle size, binder concentration and type of binder on percentage fixed carbon (PFC) the values and graph of the PFC of briquettes with 5 %, 10 % and 15 % binders (CM = 40 mesh charcoal particles with corn binder, FM = 60 mesh charcoal particles with corn binder (FM), CC = 40 mesh charcoal particles with cassava binder, FC = 60 mesh charcoal particles *with cassava binder).*

Fig. 7 shows effect of particle size, binder concentration and type of binder on percentage fixed carbon (PFC) of briquettes with 5 %, 10 % and 15 % binder contents.

The study evaluated the percentage fixed carbon (PFC) in briquette samples, revealing generally high values. As depicted in Fig. 7, the highest PFC was 70.79 % in FM samples with 5 % binder, while the lowest was 53.03 % in CC samples with 15 % binder. On average, the PFC across all binder contents were 61.45 % for CM samples, 67.54 % for FM samples, 57.58 % for CC samples, and 68.52 % for FC samples (Fig. 7). These results indicate that briquettes made from finer particles tend to have higher PFC, whereas those from coarser particles have lower PFC.

As shown in Fig. 7, PFC decreased with increasing binder content in FM, CC, and FC samples, but increased in CM samples. These findings are consistent with the understanding that finer particles improve combustion efficiency and fixed carbon content. Compared to previous studies by Duangkham et al. [26], Akintaroa et al., and Yirijor et al. [24,25], the PFC values in this work are significantly higher. The high PFC value of 70.79 % for FM samples with 5 % binder, as highlighted in Fig. 7, suggests that such briquettes are likely to be soft, lightweight, and capable of burning for an extended period. In conclusion, optimizing particle size and binder concentration is crucial for enhancing the fixed carbon content and overall performance of briquettes, contributing to more efficient and sustainable biomass fuel options.

#### 2.2.7 Effect of particle size, binder concentration and type of binder on the higher heating values of briquettes

**Fig. 8:**
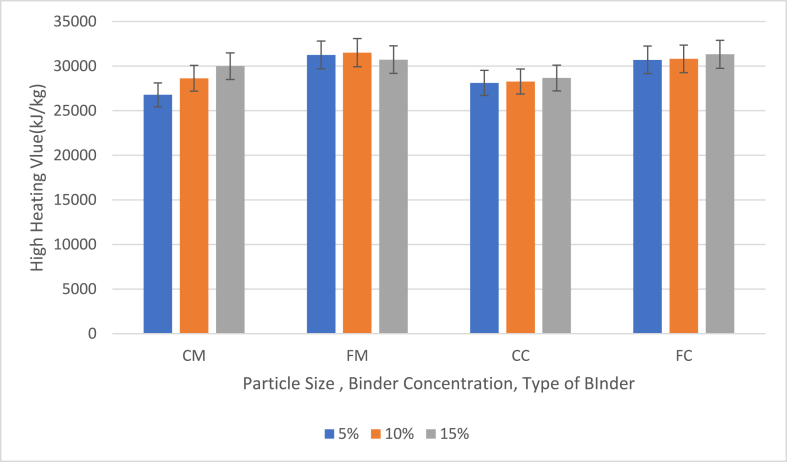
Effect of particle size, binder concentration and type of binder on the higher heating values shows the values and graph of (HHV) of briquettes with 5 %, 10 % and 15 % binders (CM = 40 mesh charcoal particles with corn binder, FM = 60 mesh charcoal particles with corn binder (FM), CC = 40 mesh charcoal particles with cassava binder, FC = 60 mesh charcoal particles with cassava binder).

Fig. 8 shows the effect of particle size, binder concentration and type of binder on the higher heating values (HHV) of briquettes with 5 %, 10 % and 15 % binder contents.

The calorific value, or energy content, of the bio-briquettes is a critical factor in determining their efficiency as a fuel source. The study revealed that the calorific values of the coconut shell charcoal-based briquettes ranged from 26,790.76 kJ/kg to 31,509.58 kJ/kg, the results of the calorific values were found to be higher than 14.1 MJ/kg obtained for maize cob briquette [36] and 18.9 MJ/kg obtained for banana peel briquette [37]. Additionally, all the briquettes produced exceed the standard minimum heating value of briquettes derived from other biomass, indicating their superior energy content and potential for efficient fuel use [38,39]. Briquettes made from coarse particles (CM and CC) showed lower HHV, whereas those made from fine particles (FM and FC) had higher HHV. The highest HHV recorded was 31,509.58 kJ/kg from FM samples with 10 % binder, and the lowest was 26,790.76 kJ/kg from CM samples with 5 % binder. Generally, the HHV increased with the binder content, except for the FM samples, which displayed some overlap. This trend can be attributed to the fact that fine particles have a higher surface area per unit mass compared to coarse particles. A higher surface area allows for better contact with oxygen during combustion, enhancing combustion efficiency. This results in a more thorough and complete burning of the briquettes.

#### 2.2.8 Effect of particle size, binder concentration and type of binder on the time taken by briquettes to boil water to 100 °C

**Fig. 9:**
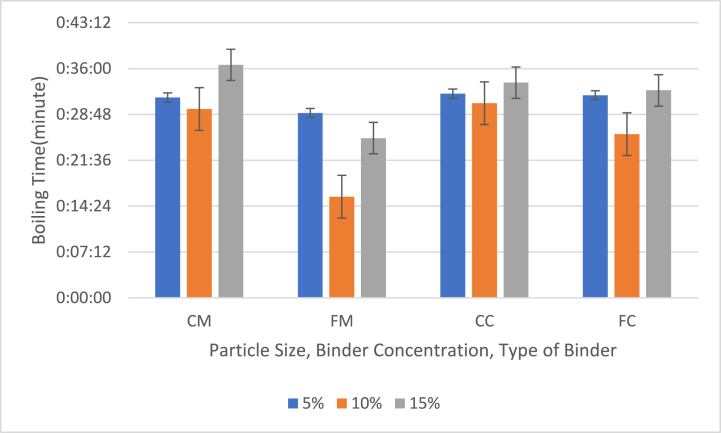
Effect of particle size, binder concentration and type of binder on the time taken by briquettes with %, 10 % and 15 % binder to boil water (CM = 40 mesh charcoal particles with corn binder, FM = 60 mesh charcoal particles with corn binder (FM), CC = 40 mesh charcoal particles with cassava binder, FC = 60 mesh charcoal particles with cassava binder).

Fig. 9 shows the effect of particle size, binder concentration and type of binder on the time taken by briquettes with %, 10 % and 15 % binder to boil water.

The boiling times for the briquette samples ranged from 15 min 53 s to 36 min 35 s. The highest boiling time was recorded for samples with 15 % coarse corn binder (CM), while the lowest boiling time was for samples with 10 % fine corn binder (FM). Generally, briquettes made from 60 mesh particles (fine) boiled water faster than those made from larger particles (coarse). Although no consistent relationship was observed between binder content and boiling time, it was noted that briquettes with 10 % binder content had the shortest boiling times.

For comparison, Sabo et al. [27] observed that corncob briquettes' temperature increased from 25 °C to 45 °C in 12 min when using a starch binder, and to 44 °C with a gum arabic binder. This faster temperature rise with starch was attributed to its lower viscosity, allowing it to burn more quickly. For coconut shell briquettes, the temperature reached 54 °C in 2 min with a starch binder and 50 °C with a gum arabic binder, and it took 10 min to boil 1 L of water [27]. These findings illustrate that the binder type and particle size significantly influence the boiling efficiency of bio-briquettes.

### 2.3 Spider web analysis

**Fig. 10:**
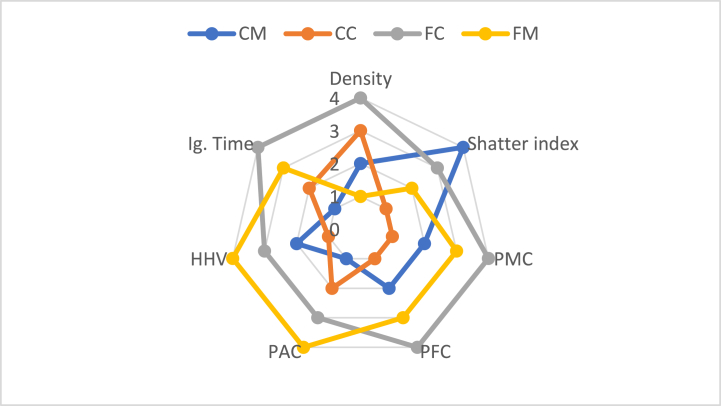
Spider-web diagram for measured properties of the briquettes.

The samples were ranked (4 points for the best value for a specific property and 1 point for the worst value) and scored for each of the measured properties, and are given in Table 1. The spider web produced from these values is shown in Fig. 10.

**Table 1:** Scoring of CM, CC, FC and FM samples with different binder contents.

Property	CM	CC	FC	FM
Density	2	3	4	1
Shatter index	4	1	3	2
Percentage moisture content	2	1	4	3
Percentage fixed carbon content	2	1	4	3
Percentage ash content	1	2	3	4
Highest heating value	2	1	3	4
Ignition Time	1	2	4	3

Fig. 10 shows that FC samples overall had the best results, although there was overlap with FM samples. Conversely, CC samples overall had the most undesirable results, although there was overlap with CM samples. Thus, samples made from fine particles generally had desirable results, whereas samples made from coarse particles had less desirable results, i.e., samples made from fine particles are best for making charcoal briquettes.

## 3 Conclusions

This study thoroughly investigated the properties of bio-briquettes made from coconut shells, focusing on various binder concentrations and particle sizes. The analysis revealed that briquettes with a 10 % fine charcoal cassava binder (FC) achieved the highest density. The finer particle size of FC allowed for better compaction and binding, resulting in more robust briquettes. In contrast, briquettes with a 15 % coarse charcoal maize (corn) binder (CM) exhibited superior shatter resistance. The larger particle size of CM contributed to a more resilient internal structure, enhancing the briquettes' ability to withstand mechanical stresses. The study also found that briquettes with a 15 % coarse charcoal cassava binder (CC) retained higher moisture and volatile matter content. This outcome is likely due to the increased binder concentration creating more voids in the briquettes, allowing for greater moisture retention and higher levels of volatile matter. Conversely, briquettes with a 5 % fine charcoal maize (corn) binder (FM) displayed higher fixed carbon content and heating values. The lower binder concentration appears to improve carbonization efficiency and reduce ash content, resulting in more efficient energy production.

Boiling tests demonstrated that briquettes with finer particle sizes, such as FC and FM, facilitated better heat transfer. The increased surface area of these finer particles likely enhanced combustion efficiency, leading to more effective heat release.These findings underscore the potential of coconut shell bio-briquettes as a viable and sustainable energy alternative. By optimizing binder ratios and particle sizes, this study provides valuable insights into enhancing both the mechanical and combustion properties of bio-briquettes. Such advancements are significant for the field of bio-briquette technology and contribute to the broader goal of utilizing agricultural waste effectively.The successful application of these bio-briquettes could significantly benefit rural communities by offering a reliable and sustainable energy source. This shift can help reduce energy poverty, minimize the environmental impact of traditional fuels, and stimulate local economic growth through new markets for agricultural by-products. Integrating bio-briquettes into Ghana's energy strategy aligns with national objectives of diversifying energy sources, improving energy security, and promoting environmental sustainability.

### 3.1 Recommendations

Based on the findings of this study, several recommendations are proposed to further enhance the effectiveness and applicability of coconut shell charcoal-based bio-briquettes.

First, there is a need to explore additional types of biomass for the production of high-quality briquettes. Given the promising results of coconut shell briquettes, researching other biomass sources could contribute to better health and environmental management by offering a broader range of sustainable energy options. This exploration could help in identifying materials that might provide even more efficient or cost-effective solutions.

Second, investigating the long-term stability of coconut shell charcoal-based bio-briquettes during storage is crucial. Future research should focus on understanding how these briquettes change over time in terms of physical and chemical properties. This includes evaluating aspects such as moisture absorption, deterioration in calorific value, and potential contamination. Such studies will be essential in determining appropriate storage conditions and establishing realistic shelf-life expectations for the briquettes, ensuring their effectiveness and safety over extended periods.

Lastly, to validate the practical applicability of these bio-briquettes, it is recommended to conduct field trials in rural communities. These trials would assess the performance and acceptability of coconut shell charcoal-based briquettes as an alternative energy source in real-world settings. Feedback from users on factors such as combustion characteristics, ease of ignition, cooking efficiency, and overall satisfaction will provide valuable insights. This practical evaluation will help in understanding how well the briquettes meet the needs of rural communities and support their integration into local energy strategies.

Implementing these recommendations will not only enhance the development and use of bio-briquettes but also contribute to more sustainable and economically beneficial energy solutions for rural areas.

## Funding statement

This research did not receive any specific grant from funding agencies in the public, commercial, or not-for-profit sectors.

## Data availability statement

The data that support the findings of this study are available from the corresponding author upon reasonable request.

## CRediT authorship contribution statement

**John Yirijor:** Writing – review & editing, Writing – original draft, Supervision, Methodology, Investigation, Formal analysis, Data curation, Conceptualization. **Alice Abigail Tatenda Bere:** Writing – review & editing, Writing – original draft, Methodology, Investigation, Data curation, Conceptualization.

## Declaration of competing interest

The authors declare no conflict of interest

## Abbreviations

**PAC**: Percentage Ash Content
**PVM**: Percentage Volatile Matter
**PFC**: Percentage Fixed Carbon
**PMC**: Percentage Moisture Content
**HHV**: Higher Heating Value
**CM**: 40 mesh charcoal particles with maize/corn binder
**FM**: 60 mesh charcoal particles with maize/corn binder
**CC**: 40 mesh charcoal particles with cassava binder
**FC**: 60 mesh charcoal particles with cassava binder
